# Effects of isoflurane anaesthesia depth and duration on renal function measured with [^99m^Tc]Tc-mercaptoacetyltriglycine SPECT in mice

**DOI:** 10.1186/s13550-023-01065-3

**Published:** 2024-01-05

**Authors:** Fabian Schmitz-Peiffer, Mathias Lukas, Ajay-Mohan Mohan, Jakob Albrecht, Jörg R. Aschenbach, Winfried Brenner, Nicola Beindorff

**Affiliations:** 1 Department of Nuclear Medicine, Charité - Universitätsmedizin Berlin, Berlin, Germany; 2 Berlin Experimental Radionuclide Imaging Center (BERIC), Charité - Universitätsmedizin Berlin, Augustenburger Platz 1, 13353 Berlin, Germany; 3 Department of Radiology, Charité - Universitätsmedizin Berlin, Berlin, Germany; 4 Institute of Veterinary Physiology, School of Veterinary Medicine, Freie Universität Berlin, Berlin, Germany

**Keywords:** [^99m^Tc]Tc-MAG3, Single mouse bed, Hotel, Isoflurane concentration, Respiratory rate

## Abstract

**Background:**

The influence of anaesthetic depth and the potential influence of different anaesthetic beds and thus different handling procedures were investigated in 86 severe combined immunodeficient (SCID) mice using semi-stationary dynamic single photon emission computed tomography (SPECT) for kidney scintigraphy. Therefore, isoflurane concentrations were adjusted using respiratory rate for low (80–90 breath/min) and deep anaesthesia (40–45 breath/min). At low anaesthesia, we additionally tested the influence of single bed versus 3-mouse bed hotel; the hotel mice were anaesthetized consecutively at ~ 30, 20, and 10 min before tracer injections for positions 1, 2, and 3, respectively. Intravenous [^99m^Tc]Tc-MAG3 injection of ~ 28 MBq was performed after SPECT start. Time-activity curves were used to calculate time-to-peak (Tmax), T50 (50% clearance) and T25 (75% clearance).

**Results:**

Low and deep anaesthesia corresponded to median isoflurane concentrations of 1.3% and 1.5%, respectively, with no significant differences in heart rate (*p* = 0.74). Low anaesthesia resulted in shorter aortic blood clearance half-life (*p* = 0.091) and increased relative renal tracer influx rate (*p* = 0.018). A tendency toward earlier Tmax occurred under low anaesthesia (*p* = 0.063) with no differences in T50 (*p* = 0.40) and T25 (*p* = 0.24). Variance increased with deep anaesthesia. Compared to single mouse scans, hotel mice in position 1 showed a delayed Tmax, T50, and T25 (*p* < 0.05 each). Furthermore, hotel mice in position 1 showed delayed Tmax versus position 3, and delayed T50 and T25 versus position 2 and 3 (*p* < 0.05 each). No difference occurred between single bed and positions 2 (*p* = 1.0) and 3 (*p* = 1.0).

**Conclusions:**

Deep anaesthesia and prolonged low anaesthesia should be avoided during renal scintigraphy because they result in prolonged blood clearance half-life, delayed renal influx and/or later Tmax. Vice versa, low anaesthesia with high respiratory rates of 80–90 rpm and short duration (≤ 20 min) should be preferred to obtain representative data with low variance.

## Introduction

Preclinical evaluation of medicinal products is an important step in the development and approval of diagnostic and therapeutic tracers. For example, the value of peptide-based tumor therapies in nuclear medicine is often compromised by increased kidney uptake and potentially toxic side effects [1–3]. Therefore, reliable and reproducible preclinical in vivo tests on kidney toxicity are of utmost importance for the establishment of new therapeutic radiopharmaceuticals in the preclinical phase.

Renal scintigraphy is a non-invasive functional imaging technique used to measure side-related glomerular filtration rate (GFR), effective renal blood flow (RBF) and blood clearance, to assess urinary tract infection, and to diagnose renal cortex scarring due to urinary tract infection. In addition, renal scintigraphy is also used in oncology for assessing treatment-induced renal function impairment. ^99m^Technetium mercaptoacetyltriglycine ([^99m^Tc]Tc-MAG3) is a tubular tracer that is commonly used for renal scintigraphy. Renal function is quantified by renal tracer uptake kinetics by calculating the effective renal plasma flow in humans [4–6], surrogated by effective RBF in mice [7], and excretion from the kidneys. Because of its robust reproducibility, renal scintigraphy is widely used in both preclinical and clinical settings. The renal [^99m^Tc]Tc-MAG3 clearance in mice has been quantified using three parameters, namely the time-to-peak uptake in the kidneys (Tmax), T50 (50% clearance), and T25 (75% clearance) [7, 8].

Renal excretion is influenced by various physiological factors such as age, sex, genetic predisposition, hydration, temperature and also circadian rhythm [7, 9, 10]. In addition to the physiological factors, it is strongly influenced by external factors such as anaesthesia. Inhalation anaesthesia results in low GFR, decreased RBF and less urine output in humans [11, 12].

Isoflurane anaesthesia is used preclinically for translational research due to its non-toxic properties and it helps minimizing stress levels during the investigations [13]. Isoflurane is widely used owing to its capability to induce immediate onset of anaesthesia in comparison with its counterparts barbiturates or ketamine and its good controllability of anaesthesia [14, 15]. It gained widespread significance in translational research as it could sustain anaesthesia over a longer period of time without multiple interventions. In radionuclide imaging, longer imaging time and immobilization is required in order to study tracer kinetics, making isoflurane the anaesthesia of choice in laboratory rodents [16, 17].

Isoflurane has been shown to affect the tracer pharmacokinetics in functional imaging. Alstrup et al. reported differential effects of isoflurane and propofol on the pharmcokinetics of radiotracers targeting dopamine receptors D1 and D5 in minipigs [18]. Another example is ^18^F-fluorodeoxyglucose ([^18^F]F-FDG), a glucose analog mapping glucose metabolism, which is widely used in oncology and neurology [19, 20]. For this tracer, an altered [^18^F]F-FDG distribution has been reported in different regions of the rat brain during isoflurane anaesthesia [21]. In nephrology, isoflurane has also profound effects on tubular functions of nephrons, which could directly affect the pharmacokinetics of [^99m^Tc]Tc-MAG3 [22].

Shikano et al. reported that the transport of [^99m^Tc]Tc-MAG3 is mediated by the organic anion transporter 1, which is expressed in the basolateral membrane of the proximal tubules of the kidney [23, 24]. The effect of isoflurane on both tubular cells and RBF could potentially affect the renal clearance of [^99m^Tc]Tc-MAG3.

Although the impact of isoflurane on various physiological mechanisms affecting renal function is well known, there is few data regarding its influence on [^99m^Tc]Tc-MAG3 excretion and renal scintigraphy. This could be a major hurdle for the adequate assessment of renal function impairment both at clinical and translational levels of drug development. Therefore, the main objective of this study was to determine renal excretion parameters under different depth levels of isoflurane anaesthesia by dynamic single photon emission computed tomography (SPECT) imaging in mice. We further assessed the effect of anaesthesia duration on renal [^99m^Tc]Tc-MAG3 kinetics by comparing anaesthesia in single beds and multiple mouse bed hotels (hotel) because renal function may be influenced by both depth and duration of anaesthesia.

## Material and methods

Due to the importance of severe combined immunodeficient (SCID) mice as a tumor model in oncology, they were used for all experiments in this study. Animals were housed under standardized conditions with a light–dark cycle following a 12-h rhythm. Further details of the animal husbandry are described elsewhere [25].

Renal scintigraphy was performed with combined SPECT and computed tomography (CT) using the NanoSPECT/CTplus (Mediso, Hungary /Bioscan, France). Each detector was equipped with a nine pinhole mouse aperture for high resolution (*d* = 1.0 mm) with a spatial resolution of 0.7 mm full-width-at-half-maximum (FWHM) for ^99m^Tc when using a single mouse bed or a high resolution rat aperture (*d* = 1.5 mm) with a spatial resolution of 1.16 mm FWHM [26] when using the hotel.

**Fig. 1:**
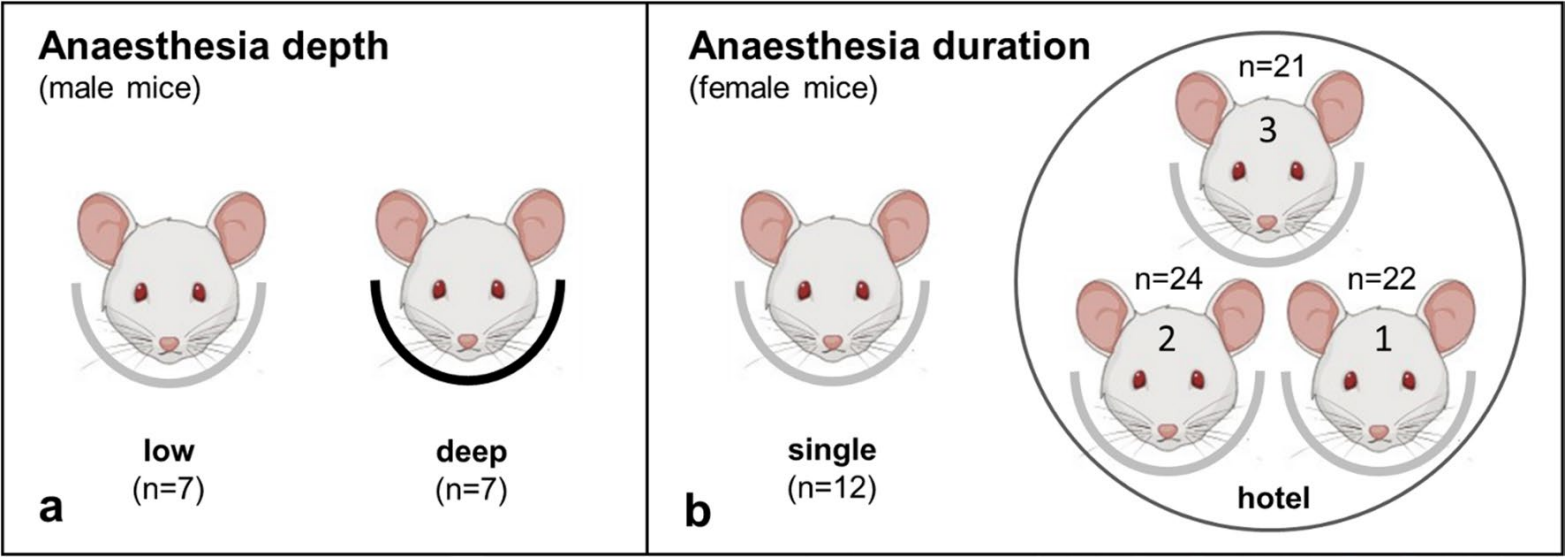
**a** Investigation of isoflurane anaesthesia depth controlled by respiratory rates (low = 80–90/min, deep = 40–45/min) in a single mouse bed. [^99m^Tc]Tc-MAG3 injection started approx. 10 min after anaesthesia induction. **b** Investigation of isoflurane anaesthesia duration at low anaesthesia in comparison between mice imaged in a single mouse bed and in a 3-mouse bed hotel. At the time of tracer injection, anaesthesia duration was approx. 30 min for mice in hotel position 1, 20 min in position 2, and 10 min in position 3 and single mouse bed. Mice created with Biorender.com

Figure 1 shows the study design for the experiments on the influence of anaesthesia depth (Fig. 1a) and duration (Fig. 1b) in mice, which is described in the following subsections.

### Investigation of isoflurane anaesthesia depth on renal function

Seven male SCID mice aged 5 months with a median bodyweight of 25.0 g (23.4–27.0) underwent renal scintigraphy. In order to evaluate the effects of anaesthesia depth on murine renal function, imaging was performed under low and high isoflurane concentration with an interval of 7 days in between. Mice were anaesthetized with 1–2% isoflurane with oxygen at a flow rate of 0.8 l/min and placed in prone position on a heated (37 °C) single mouse bed (Equipement Vétérinaire Minerve, France) in the scanner. A respiratory pillow was used to monitor respiratory rate for anaesthesia adjustment throughout image acquisition. Since mice react differently to the same anaesthesia concentration, the depth of anaesthesia was controlled as a function of the respiratory rate. Respiratory rates of 80–90/min and 40–45/min were used for adjust to a low and deep anaesthesia, respectively. Additionally, neonatal monitoring electrodes for electrocardiography (ECG; Red Dot™, 3 M Health Care, Neuss, Germany) were fixed to the paws.

To avoid possible bias due to carry-over effects from the previous anaesthesia, half of the animals were first examined at low isoflurane concentration and then at high concentration; while, the examinations in the other half started with initial examinations at high isoflurane concentration. Although Huang et al. [7] demonstrated that circadian rhythm has no significant effect on renal function in mice, in the same animal in the present study the two follow-up studies were performed at the same time of day to exclude possible effects of circadian rhythm on anaesthetic outcomes.

### Investigation of isoflurane anaesthesia duration on renal function at low anaesthesia

Because female mice have a later renal Tmax after 3 months of age in contrast to males [7], the comparison between single and multiple beds was examined in mice of the same sex.

Twelve female SCID mice, aged 3 months with a median body weight of 19.6 g (16.5–26.4), underwent renal scintigraphy in a heated single mouse bed (Equipement Vétérinaire Minerve), and each detector was equipped with a high resolution mouse aperture. A respiratory rate of 80–90 breaths/min was maintained throughout the investigation. Tracer injection immediately followed SPECT start at approx. 10 min after anaesthesia induction.

Another 67 female SCID mice age 3–4 months with a median body weight of 20.3 g (15.5–24.3) underwent renal scintigraphy in a heated 3-mouse bed hotel (Equipement Vétérinaire Minerve), providing 21–24 mice per bed position. Due to the hotel size, detectors were equipped with high resolution rat apertures. Measurement of isoflurane concentration for the individual mouthpieces in the hotel proved a uniform concentration distribution with less than ≤ 0.01% difference in isoflurane concentration between the three bed positions (MultiGasAnalyser OR-703/FlowAnalyser PE-300, IMT Analytics, Switzerland; accuracy 0.15 vol% + 5% of reading). A maximum isoflurane concentration of 1.2% was maintained as respiration monitoring was not feasible in the hotel. After the first mouse had been fitted with an intravenous tail catheter for tracer injection, it was placed in the hotel. Then the 2nd and 3rd mouse was also prepared for scintigraphy with approx. 10 min time difference between mice. Tracer injection after SPECT start was performed in the three mice in the same order as catheter placement, with an interval of approx. 30 s. Thus, at the time of tracer injection, duration of anaesthesia was approx. 30 min for mice in position 1, 20 min in position 2, and 10 min in position 3.

### Renal scintigraphy by semi-stationary SPECT/CT

The mice were under isoflurane anaesthesia during catheterization, tracer injection and the entire SPECT/CT acquisition. Intravenous tail injection of [^99m^Tc]Tc-MAG3 was carried out with a 30 G cannula and a 0.28 × 0.61 mm catheter of 40 cm length (A. Hartenstein, Portex, Germany) filled with 2 I.U. heparin (Medunasal®) per ml 0.9% NaCl, as described by Huang et al. [7]. The kidneys were positioned in the scan range for semi-stationary SPECT by a low-dose CT scan (one bed position only with 14 mm for high resolution mouse apertures and 22 mm for high resolution rat apertures). Thereafter, SPECT acquisition was started directly before the intravenous injection of approx. 28 MBq [^99m^Tc]Tc-MAG3 in a maximum volume of 0.2 ml including flushing the catheter. The semi-stationary dynamic SPECT acquisition with intersampling was performed with two detector positions per frame displaced by 45°, and consisted of 10 × 20 s frames (10 s per detector position) followed by 25 × 50 s frames (25 s per detector position) creating a total of 68 reconstructed images. The total duration of the scanning procedure was 35 min. As the three mice in the hotel were injected consecutively after SPECT start, the dynamic data set started with time frames of 15 × 20 s to ensure the detection of Tmax of the 3rd mouse during the 20 s frames as well. This resulted in a total of 78 reconstructed images over a period of 38 min. The total duration of anaesthesia of an animal depended on the time of catheterization and the time until the SPECT acquisition started. This additional anaesthesia time was 10 min for the mice in the single mouse bed and in hotel position 3. In contrast, the additional anaesthesia time for the mice that were first placed in the hotel (position 1) was approx. 30 min, and approx. 20 min in position 2. After completion of the SPECT acquisition, tail catheters were removed and animals were placed in a heating box to wake up before being returned to their respective animal group.

### Quantification and statistical analysis

The SPECT device is regularly calibrated by the manufacturer, including high voltage, energy and count rate linearity, energy resolution and spatial uniformity using point sources and different phantoms. Finally, the SPECT system is cross-calibrated with a dose calibrator for each nuclide by imaging a defined amount of radioactivity within either mouse or rat phantoms, depending on the imaging task. This generates absolute values in kBq for each calibrated nuclide with sufficient quantitative accuracy [27].

**Fig. 2:**
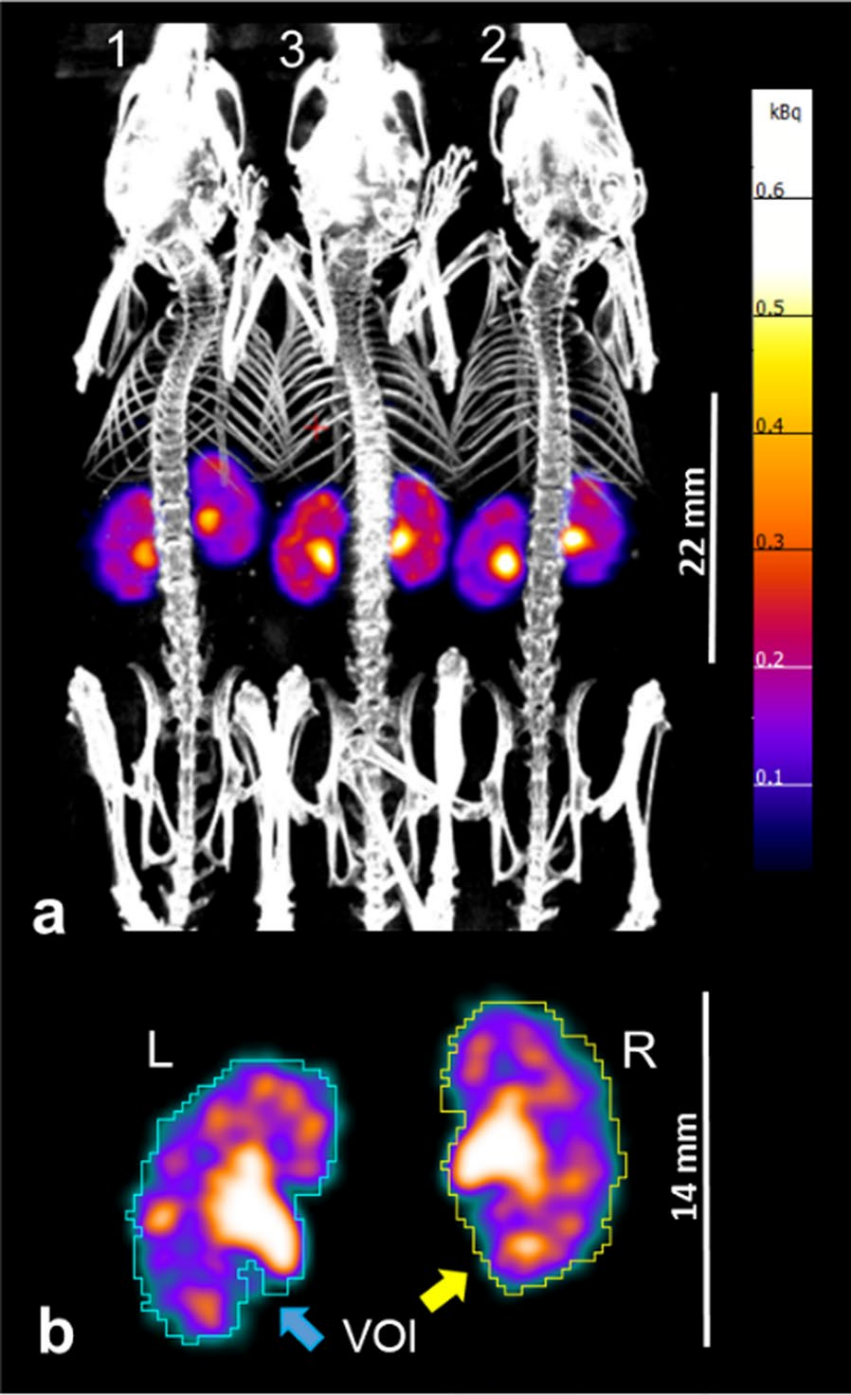
**a** Maximum intensity projection of a semi-stationary dynamic SPECT acquisition fused with whole-body CT of mice acquired in a 3-mouse bed hotel with 22 mm scan range (high resolution rat apertures) after intravenous injection of [^99m^Tc]Tc-MAG3. The color bar represents kBq. **b** SPECT acquisition in a single mouse bed with 14 mm scan range (high resolution mouse apertures) and manual contouring of a volume-of-interest (VOI) to quantify [^99m^Tc]Tc-MAG3 uptake

Aortic and renal uptake kinetics of [^99m^Tc]Tc-MAG3 was evaluated by windowing the data to 0%-40% of the maximal activity in the time frame with highest uptake (approx. frame 6) and defining a volume-of-interest (VOI) with an isocontour of 1.0% threshold (refers to metric = (maximum-minimum of voxel values)) using PMOD 3.5 (PMOD Technologies Ltd., Switzerland). Contouring a VOI for each kidney included renal cortex, medulla and the pelvicalyceal system as shown in Fig. 2b. The time activity curve of each kidney was obtained by plotting the absolute measured activity values of each VOI against time. Time-to-peak (Tmax), T50 (50% clearance) and T25 (75% clearance) and blood excretion half-life (aorta 50% clearance) were calculated and used for further statistical analysis as described previously [7].

Data are presented as median, interquartile range [IQR, 25th–75th percentile], minimum and maximum and pictured as boxplots. The comparison of paired groups (i.e., low vs. deep anaesthesia) was performed with the Wilcoxon test whereas unpaired groups (i.e., single bed vs. hotel) were compared with the Mann–Whitney U test. Significance was assumed at *p* < 0.05.

Due to the low total blood volume of a mouse, repeated blood sampling to determine serum creatinine (clearance) and other physiological renal blood parameters was not possible in this longitudinal study.

## Results

Since the right kidney in mice is located further cranially than the left kidney, placing both kidneys is difficult or often insufficient when using mouse apertures with only 14 mm scan range. Consequently, rat apertures with a larger transaxial field of view of 22 mm are suitable for renal scintigraphy with semi-stationary SPECT in mice. Figure 2 shows the use of two different apertures (Fig. 2a rat aperture, Fig. 2b mouse aperture) and that resolution of the images is comparable.

### Effects of isoflurane anaesthesia depth on renal function

A summary of different parameters during renal scintigraphy under the influence of the depth of anaesthesia is given in Table 1. The depth of anaesthesia in this study was regulated on the basis of the respiratory rate, instead of isoflurane concentration. Correspondingly, a significantly different respiratory rate (89 [87–90] 81–92 versus 47 [44–48] 43–51; *p* = 0.018) was obtained for respiratory monitoring.

**Table 1:** Different parameters during renal [^99m^Tc]Tc-MAG3 scintigraphy in single mouse bed examinations as influenced by the depth of anaesthesia controlled by respiratory rate

	Low anaesthesia Respiratory rate: 80–90/min (n = 7)	Deep anaesthesia Respiratory rate: 40–45/min (n = 7)	*p* value
Isoflurane (%)	1.3 [1.1–1.5] 0.8–1.5	1.5 [1.5–1.7] 1.2–1.9	0.078
Respiratory rate (breaths/min)	89 [87–90] 81–92	47 [44–48] 43–51	0.018
Heart rate (beats/min)	328 [326–336] 290–365	343 [307–356] 281–369	0.74
Relative renal tracer influx rate (MBq/ml/min)	6.0 [5.1–6.2] 3.5–6.5	4.1 [3.4–4.3] 2.8–4.9	0.018
Aorta excretion (seconds)			
T50aorta	29 [28-36] 25–40	35 [32-37] 28–42	0.091
Kidney uptake (min)			
Tmax	1.5 [1.5–1.5] 1.3–1.8	1.8 [1.6–2.2] 1.4–2.9	0.063
T50	4.5 [4.1–4.7] 3.5–4.9	4.1 [4.0–5.8] 3.6–6.7	0.40
T25	7.0 [6.5–8.7] 6.1–9.2	8.6 [7.4–10.5] 7.2–12.1	0.24

Aorta tracer excretion half-life is expressed as T50aorta in seconds and kidney uptake as time-to-peak (Tmax), T50 (50% clearance) and T25 (75% clearance) in minutes. Each set of data includes the median, interquartile range [IQR], min–max and number of animals

In comparison, only a non-significant trend (*p* = 0.078) was observed for isoflurane concentration as a function of depth of anaesthesia with a correlation factor rho = −0.54 (*p* = 0.049).

Heart rate monitoring showed no significant difference (*p* = 0.74) between the two anaesthetic regimes (rho = 0.18, *p* = 0.54). This was also reflected in the low correlation between heart rate and respiratory rate (rho = −0.02, *p* = 0.94).

Low anaesthesia with high respiratory rate resulted in a tendency for shorter blood clearance half-life (T50aorta, *p* = 0.091) and significantly increased relative renal tracer influx rate (*p* = 0.018). However, as a function of heart rate, blood clearance half-life (rho = −0.63, *p* = 0.015) and relative renal tracer influx rate (rho = −0.50, *p* = 0.071) accelerated with increasing heart rate.

**Fig. 3:**
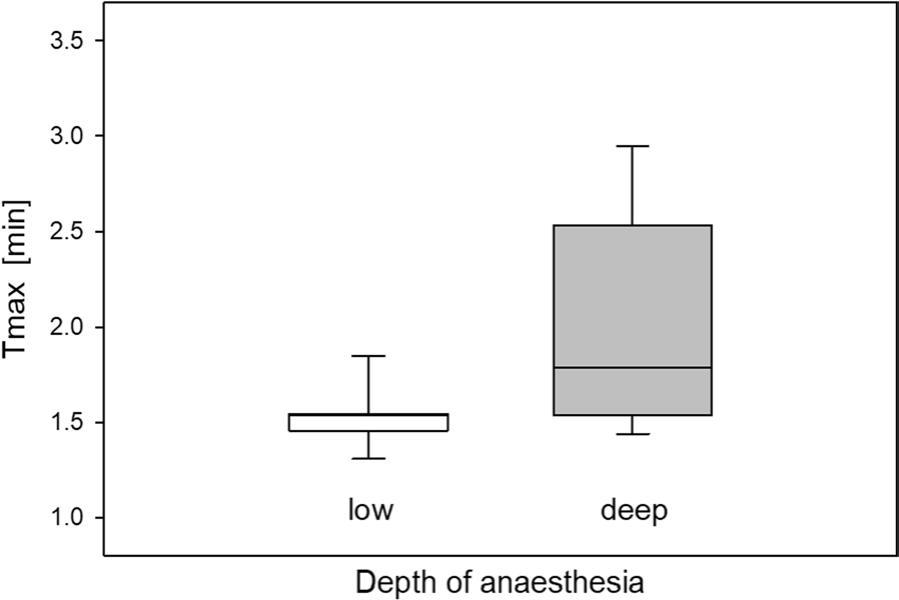
Time-to-peak (Tmax) of [^99m^Tc]Tc-MAG3 kidney uptake during low and deep anaesthesia in single mouse bed examinations (*p* = 0.063)

Kidney [^99m^Tc]Tc-MAG3 uptake kinetics showed a tendency for an earlier time-to-peak (Tmax) under low anaesthesia (*p* = 0.063). In comparison, T50 (*p* = 0.40) and T25 (*p* = 0.24) did not show any significant difference depending on the depth of anaesthesia. Data variance increased during deep anaesthesia, which can be seen in Fig. 3. Heart rate had no impact on [^99m^Tc]Tc-MAG3 uptake kinetics (Tmax: rho = 0.04, *p* = 0.16; T50: rho = −0.04, *p* = 0.91; T25: rho = −0.22, *p* = 0.45).

### Effects of single or multiple mouse beds on renal function

**Fig. 4:**
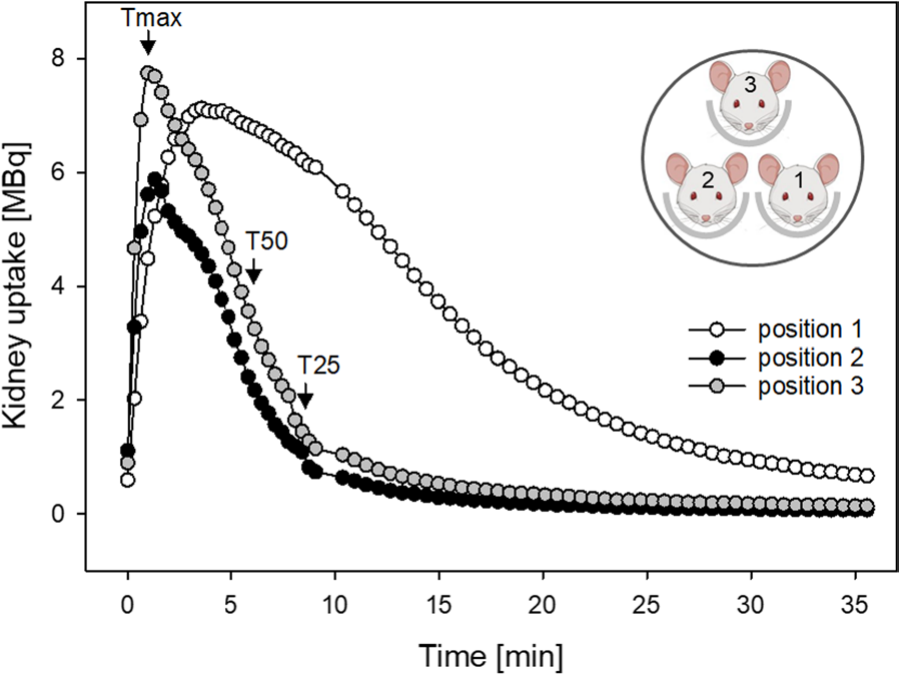
Example of [^99m^Tc]Tc-MAG3 time activity curves of renal function (left and right kidney pooled, respectively) from three mice imaged simultaneously within a 3-mouse bed hotel after intravenous tracer injection. At time of tracer injection, duration of anaesthesia was approx. 30 min for position 1, 20 min for position 2, and 10 min for position 3. The mouse in position 1 showed delayed time-to-peak (Tmax), T50 (50% clearance) and T25 (75% clearance). Mice created with Biorender.com

Figure 1b and Fig. 2a show the positions 1–3 of individual mice in the hotel. Mice in position 1 were under anaesthesia longest (approx. 30 min) and in position 3 shortest (approx. 10 min) until the onset of scintigraphy. An influence of the hotel position is clearly shown for mice in position 1 by a significantly delayed Tmax, T50 and T25 in comparison with mice in positions 2 and 3 as well as in comparison with the mouse in single bed (Table 2). In contrast, no difference could be found between the single bed examination and positions 2 or 3 in the hotel (*p* = 1.0 each). When comparing the different positions in the hotel alone, there was a significant difference between position 1 and position 2 (delayed T50 and T25; *p* < 0.01 each), as well as between position 1 and position 3 (delayed Tmax, T50, and T25; *p* < 0.05 each). Figure 4 shows the time activity curves of renal function of three mice of the same acquisition. The mouse in position 1 had an obvious delay in Tmax.

**Table 2:** [^99m^Tc]Tc-MAG3 kidney kinetics compared between single mouse bed and 3-mouse bed hotel examinations

	Single	Position 1	Position 2	Position 3
Aorta excretion (seconds)				
T_50_aorta	64.9 #	28.2#	31.4	44.2
	[32.1–179.6]	[21.2–53.1]	[22.9–61.0]	[25.8–66.4]
	21.5–246.9	10.7–68.5	10.3–237.1	17.1–111.1
No. of animals	12	22	24	21
Kidney uptake (min)				
Tmax	1.4 #	1.8 # ♦	1.5	1.4 ♦
	[1.3–1.5]	[1.6–2.1]	[1.3–1.9]	[1.1–2.2]
	1.2–5.6	1.3–5.6	1.2–2.3	1.1–2.9
T50	3.8 #	5.3 # + ♦	3.7 +	4.1 ♦
	[3.5–4.3]	[4.1–7.1]	[3.3–4.7]	[3.2–5.1]
	3.1–4.9	3.3–15.3	2.3–7.9	2.3–6.9
T25	6.5 #	9.2 # + ♦	5.9 +	6.6 ♦
	[5.8–7.3]	[6.8–11.3]	[5.2–7.8]	[5.1–8.5]
	4.9–7.8	5.3–26.3	3.6–13.2	2.5–10.9
No. of animals	12	22	24	21

Aorta tracer excretion half-life is expressed as T50aorta in seconds and kidney uptake as time-to-peak (Tmax), T50 (50% clearance) and T25 (75% clearance) in minutes. Each set of data includes the median, interquartile range [IQR], min–max and number of animals

^**#**^*p* < 0.05 between single versus position 1

**+***p* < 0.01 between position 1 versus 2

♦*p* < 0.05 between position 1 versus 3

## Discussion

As mentioned in the introduction, preclinical studies are an important step in translational research in which new target molecules are tested for their effect. Inhalation anaesthesia is not only used in preclinical radionuclide imaging, but also in various other preclinical analyses. Therefore, it is extremely important for drug development, biodistribution studies and application to evaluate the effects of isoflurane in experimental animals to enable accurate translational research. Inhalational anaesthesia is known to impair cognitive functions such as learning and memory in neonates, humans and mice [28, 29]. Radionuclide imaging has also shown altered [^18^F]F-FDG distribution in different brain regions of the rat under anaesthesia compared to consciousness [21]. Similarly, Li et al. reported that prolonged exposure to 1% isoflurane for four hours impairs brain function and ultimately MRI brain imaging parameters in both mice and humans [30].

Anaesthesia can indirectly affect renal function through influences on hemodynamics, sympathetic activity and humoral regulation, which may be more pronounced than the direct effects on the kidney. Direct effects, which are dose- and drug-dependent, include effects on autoregulation of RBF, alterations of antidiuretic hormone action and tubular transport of sodium and organic acids [11]. Given the wide-ranging effects of anaesthesia on renal function and other physiological processes with impact on RBF [31, 32], this study focused on the role of the widely used preclinical inhalational anaesthetic isoflurane on [^99m^Tc]Tc-MAG3 kinetics and kidney scintigraphy.

A dose-dependent respiratory depression and an increase in respiratory rate in humans was observed when isoflurane concentration was increased [33]. Murat et al., however, reported no change in respiratory rate with different isoflurane concentrations in children [34]. In the present study, the depth of anaesthesia was adjusted on the basis of respiratory rate. There was a significantly reduced respiratory rate with deep anaesthesia compared to a lower depth of anaesthesia in SCID mice. However, a high variability in individual animal-specific susceptibility to isoflurane was indicated because only a tendency for a higher isoflurane concentration could be found for deep vs. low anaesthesia.

Isoflurane has been shown to have a major impact on the cardiovascular system, mainly, by suppressing the sympathetic nervous system. Thereby, isoflurane anaesthesia commonly decreases blood pressure with increasing depth of anaesthesia as shown for, e.g., rabbits [35], cats [36] and rats [37]. The heart rate response, however, appears to differ. In rabbits [35] and cats [36], isoflurane induced tachycardia that appeared mainly attributable to an additional vagal withdrawal [35]. In rats, Albrecht et al. also observed a significant increase in heart rate under 2–4% isoflurane [38]. However, Yang et al. observed a significant decrease in heart rate, blood pressure and cardiac function in rats when isoflurane concentrations increased from 0.5 to 1%, 1.5%, 2% and 3% [39]. Although there was a slight increase in heart rate from 0.5 to 1% isoflurane, this increase was not significant [39]. Similar to our current study, Lee et al. found no changes in heart rate at 1% and 2% isoflurane anaesthesia in rats and explained this with an absent vagal withdrawal and a concurrently blunted baroreceptor reflex [37]. Thus, the degree of vagal depression appears to be decisive for increases vs. decreases or non-effects on heart rate during isoflurane anaesthesia.

Following isoflurane induction in humans, there was a 30–50% reduction in RBF, 50% reduction in GFR [40, 41] and total urine excretion [11]. In sheep, where RBF was measured directly, a reduction was also observed under isoflurane [42]. A reduction in RBF by isoflurane was associated with renal hypoxia and increased renal sympathetic nerve activity (RSNA) in sheep [43, 44]. Sevoflurane also decreased RBF and increased RSNA; while, renal denervation normalized RBF, suggesting that at least part of the RBF reduction during sevoflurane anaesthesia was due to RSNA-induced renal vasoconstriction [45]. Consistent with those findings of the above mentioned literature, deep anaesthesia resulted in a prolonged blood clearance half-life and a significantly decreased relative renal tracer influx rate in the present mouse study.

Another important factor responsible for decreased renal function is thermoregulation. Hypothermia causes vasoconstriction, thereby, restricting the blood flow to organs to conserve heat. Reduction of the core temperature to 25 °C in chloralose/urethane anaesthetized rats reduced heart rate, arterial blood pressure and GFR [46]. The effects of hypothermia at 28 °C body temperature for 1–2 h in another rat study showed that RBF decreased but systemic blood pressure remained essentially unchanged under these conditions [10]. The RBF reduction observed during hypothermia was due to a 75% increase in vascular resistance caused mainly by constriction of afferent arterioles and increased blood viscosity. This was accompanied by a decrease in glomerular capillary pressure and a decrease in GFR [10]. Anaesthetic beds cannot be adequately closed during renal scintigraphy due to animal handling and indwelling catheters placed in the tail vein. The first placed and longest anaesthetized mouse therefore might adapt its thermoregulation to lower ambient temperatures despite the bed heated at 37 °C. Albrecht et al. reported a drop in blood temperature to 36.6 °C in rats during the sixth isoflurane anaesthesia of 40 min duration within three weeks [38]. Therefore, it might be possible that a slight cooling of animals with time promoted vasoconstriction and partly contributed to a reduced RBF. In such scenario, GFR is maintained for a time due to the Bayliss effect and tubular–glomerular feedback; however, RBF and thus peritubular perfusion may decrease, which impedes tubular secretory output and thus [^99m^Tc]Tc-MAG3 kinetics.

Accordingly, [^99m^Tc]Tc-MAG3 kidney uptake showed a tendency toward a later Tmax during deep isoflurane anaesthesia compared to low anaesthesia in the present study. Even more pronounced were the effects of anaesthesia duration on [^99m^Tc]Tc-MAG3 kinetics for mice in position 1 in our 3-bed hotel that were anaesthetized longest due to catheter preparation and placement in the anaesthetic bed; these mice showed significantly delayed Tmax, T50 and T25 compared to mice in position 2 or 3 with a much shorter anaesthesia time of only 10 or 20 min until tracer injection. No difference occurred between single bed examination and positions 2 or 3 in the hotel. These findings are of high practical relevance because the use of multiple-bed hotels is attractive in nuclear medicine for studying as many animals as possible with one synthesis of tracers [47]. Our results clearly demonstrate that prolonged anaesthesia has a negative effect on renal kinetics. Prolonged anaesthesia could be avoided by parallel anaesthesia induction and catheterization of several mice in the hotel. However, this cannot usually be guaranteed due to the staffing situation in the laboratories.

Another point of interest in multiple-bed hotels besides length of anaesthesia is the potentially varying depth of anaesthesia of different animals. Even if the respiratory rate can be individually monitored in state-of-the art manufactured new hotels, the anaesthesia of mice cannot usually be adjusted individually per bed position with regard to the individual respiratory rate; and thus, all animals are receiving the same isoflurane concentration. Since our results indicate a highly individual animal-specific susceptibility to isoflurane, this will result in different individual anaesthesia depths of different animals with additional impact on individual [^99m^Tc]Tc-MAG3 kinetics. Therefore, based on our findings, the use of multiple-bed hotels has clear limitations, and if necessary, it should be limited to two mice per hotel.

## Conclusion

Deep anaesthesia with decreased respiratory rate in mice resulted in prolonged blood clearance half-life, delayed relative renal tracer influx rate, later Tmax and increased kinetic variance. Therefore, respiratory rates should be kept constant between individuals and low depth of anaesthesia with high respiratory rates of 80–90 rpm should be preferred during renal scintigraphy to obtain representative data with low kinetic variance.

A longer duration of low anaesthesia, as experienced by the mouse placed first in a 3-bed hotel, has also negative influences on the renal parameters. To avoid prolonged anaesthesia, investigations in multiple mouse bed hotels should be limited to a maximum of two simultaneously examined mice, which still suffers from the impossibility for individual adjustment to low anaesthesia.

In addition to the reproducibility of renal scintigraphy results, a low depth and short duration of anaesthesia should also be considered as a critical refinement option for the experimental animal.

## Data Availability

The datasets used and analyzed during the current study are available from the corresponding author on reasonable request.

## Abbreviations

CT: Computed tomography
ECG: Electrocardiography
[^18^F]F-FDG: ^18^F-fluorodeoxyglucose
FWHM: Full-width-at-half-maximum
GFR: Glomerular filtration rate
IQR: Interquartile range [25th–75th percentile]
LAGeSo: Landesamt für Gesundheit und Soziales, Berlin
RBF: Renal blood flow
RSNA: Renal sympathetic nerve activity
SCID: Severe combined immunodeficient
SPECT: Single photon emission computed tomography
[^99m^Tc]Tc-MAG3: ^99m^Technetium mercaptoacetyltriglycine
Tmax: Time-to-peak
T50: 50% Clearance
T25: 75% Clearance
VOI: Volume-of-interest
